# siRNA-mediated targeting of the RNA-dependent RNA polymerase in the Norovirus genome

**DOI:** 10.1186/1753-6561-9-S7-A11

**Published:** 2015-10-27

**Authors:** Rachel Mac Cann, Sam McConkey, Fiona O'Brien

**Affiliations:** 1 Royal College of Surgeons in Ireland, Dublin, Ireland

## Background

Human noroviruses are a major cause of epidemic and sporadic gastroenteritis worldwide and can chronically infect immunocompromised patients. Efforts to develop effective vaccines and antivirals have been hindered by the uncultivable nature and extreme genetic diversity of human noroviruses [1]. Previous therapies have focused on targeting nucleases and structural proteins. The norovirus RNA genome or viral transcripts also constitute an important target to inhibit the replication of norovirus. Finding a conserved component of the Norovirus genome would make it an ideal target that could overcome the genetic diversity of strains [2].

Gene-based therapy is the intentional modulation of gene expression in specific cells to treat pathological conditions. This modulation is accomplished by introducing exogenous nucleic acids such as small interfering RNA (siRNA). Given the large size and negative charge of this macromolecule, its delivery is typically mediated by carriers or vectors. Targeting the gastrointestinal (GI) tract represents a promising strategy for local or systemic delivery of gene-based therapeutics [3].

## Methods

A common Norovirus Genotype to the Irish population was identified. Conserved components of the Norovirus genome were isolated for targeted antiviral therapy. BLAST searches and homology searches were conducted on these sequences identified.

A Literature review was conducted on siRNA-mediated inhibition techniques and its application to the gastrointestinal tract. A review was also conducted on the recent advances in material sciences, nanotechnology and nucleic acid chemistry that have yielded promising non-viral delivery systems.

## Results

The Sydney 2012 genotype was found to be the most prevalent virus strain in Ireland and the RNA dependant RNA polymerase (RdRp) was found to be a conserved component. Two candidate sequences of the RdRP were identified for siRNA mediated inhibition.

## Conclusions

The development of a Nano-carrier for siRNA delivery via the GI tract would enable localised therapy for Norovirus. Gene therapy via this route has many advantages, including non-invasive access and the versatility to treat local diseases, such as Norovirus. However, the intestine presents several distinct barriers and, therefore, the design of robust non-viral delivery systems is key to future success. Several non-viral delivery strategies have provided evidence of activity in vivo [4,5]. This report reviews the possibility of siRNA-mediated inhibition of the RNA norovirus genome and the challenges that an oral gastrointestinal-delivery system will face.
